# Anti-Inflammation Property of *Syzygium cumini* (L.) Skeels on Indomethacin-Induced Acute Gastric Ulceration

**DOI:** 10.1155/2015/343642

**Published:** 2015-11-08

**Authors:** Lanchakon Chanudom, Jitbanjong Tangpong

**Affiliations:** Biomedical Sciences, School of Allied Health Sciences and Public Health, Walailak University, Nakhon Si Thammarat 80160, Thailand

## Abstract

Indomethacin, nonsteroidal anti-inflammatory drug (NSAIDs), induced gastric damage and perforation through the excess generation of reactive oxygen species (ROS). *Syzygium cumini* (L.) Skeels is commonly used as a medicinal plant and is claimed to have antioxidant activities. The effects of *Syzygium cumini* (L.) Skeels aqueous extract (SCC) on antifree radical, anti-inflammation, and antiulcer of SCC on indomethacin induced acute gastric ulceration were determined in our study. Scavenging activity at 50% of SCC is higher than ascorbic acid in *in vitro* study. Mice treated with indomethacin revealed mucosal hemorrhagic lesion and inhibited mucus content. Pretreatment with SCC caused discernible decrease in indomethacin induced gastric lesion and lipid peroxide content. In addition, oxidized glutathione (GSSG), glutathione peroxidase (GPx), nitric oxide (NO) levels, and gastric wall mucus were restored on acute treated mice model. Indomethacin induced inflammation by activated inducible nitric oxide synthase (iNOS) and tumor necrosis factor-alpha (TNF-*α*) proinflammatory cytokines to release large amount of ROS/RNS which were ameliorated in mice pretreatment with SCC. SCC showed restoration of the imbalance of oxidative damage leading to amelioration of cyclooxygenase enzyme (COX). In conclusion, SCC acts as an antioxidant, anti-inflammation, and antiulcer against indomethacin.

## 1. Introduction

*Syzygium cumini* (L.) Skeels from Myrtaceae family is a native tree of the tropics, originally from India and Southeast Asia. It is widespread in north, northeast, and south of Thailand and used as a popular treatment against various diseases. The bark, fruits, seeds, and leaves of* Syzygium cumini* (L.) Skeels were used for the treatment of diabetes and administered in various pharmaceutical preparations in Brazil [1]. Seeds have shown hypoglycemic and antioxidant activities. The bark is also used for dysentery and diarrhea. Moreover,* Syzygium cumini* (L.) Skeels has been shown to have sedative and anticonvulsant effects and a potent central nervous system depressant effect [2] and this plant is rich in compounds containing anthocyanins, glucoside, ellagic acid, isoquercetin, kaempferol, and myricetin. The leaves are claimed to contain acylated flavonol glycosides, quercetin, myricetin, myricetin 3-0-4-acetyl-L-rhamnopyranoside, triterpenoids, esterase, galloyl carboxylase, and tannin [3].

Among the commonly used nonsteroidal anti-inflammatory drugs (NSAIDs), indomethacin possesses the highest ulcerogenic potential to humans. Nevertheless, it is still extensively used in obstetrics to delay uterine contractions and in the neonatal unit to facilitate patent ductus arteriosus closure. Also, it is most preferred for various mechanistic studies on NSAID physiology. Inhibition of the cyclooxygenases (COXs) and the associated reduced prostaglandin (PG) synthesis were believed to be the major reasons for gastric pathogenesis caused by NSAIDs including indomethacin. However, accumulated evidence suggested that multiple other COX-independent factors play equally important roles in the process. Indomethacin is a known inducer of reactive oxygen and nitrogen species in animal models [4], which may contribute to mucosal injury. It is becoming increasingly apparent that leukocyte-endothelial cell interaction, caused by various adhesion molecules [5], is a critical and early event in the pathogenesis of NSAID-induced gastropathy.

The treatment of ulceration on NSAIDs induced gastric damage used the existing synthetic antiulcer drugs which confer mild-to-severe side effects and are expensive especially for the rural people and some of the antiulcer drugs are known to exert their action via antioxidative activity [6]. Therefore, the traditional plants which have high antioxidants actions might provide inexpensive and nontoxic medications for people. To find out this problem, our research was designed and aimed to determine the total phenolic and flavonoid contents, scavenging capacities in cell free system, anti-inflammation, and antiulcer action against indomethacin NSAIDs induced gastric damage of* Syzygium cumini* (L.) Skeels aqueous crude extracts (SCC). Thus, our experiment may highlight the potential of SCC as a new source of natural antioxidants and anti-inflammation and as a peptic ulceration inhibitor.

## 2. Materials and Methods

### 2.1. Plants

*Syzygium cumini* (L.) Skeels was collected from different natural habitats areas at Nakhon Si Thammarat province, Thailand. The fresh leaves were rinsed with distilled water. To prepare aqueous leaf extracts of* Syzygium cumini* (L.) Skeels or SCC,* Syzygium cumini* (L.) Skeels was chopped and homogenized into distilled water in a blender for 1 min. After that the suspension was filtered by Whatman number 1 and then lyophilized by freeze dryer at −20°C for 20 h. The powder was kept at −30°C until used.

### 2.2. Determination of Total Phenolic and Flavonoid Contents of SCC

The total phenolic of SCC was determined with the Folin-Ciocalteu reagent using the method of Lister and Wilson [7] as slightly modified on this study. Briefly, 20 *µ*L of sample in triplicate and 100 *µ*L of 2 N Folin-Ciocalteu's reagent were added and incubated at room temperature for 5 min. 300 *µ*L of  Na_2_CO_3_ (25% w/v) was mixed and incubated at 45°C for 30 min. The absorbance of SCC was read at 765 nm using UV-visible spectrophotometer. Results were expressed as microgram of gallic acid equivalent per milliliter (mgGAE/mL).

Aluminum chloride colorimetric method was used for flavonoid determination [8]. 1 mL of SCC (100 *µ*g/mL) was mixed with 3 mL of methanol, 0.2 mL of 10% AlCl_3_, 0.2 mL of 1 M C_2_H_3_KO_2_, and 5.6 mL of distilled water and then incubated at room temperature for 30 min and the absorbance of the reaction mixture was measured at 415 nm with UV-visible spectrophotometer. The calibration curve was prepared by preparing quercetin solutions at various concentrations in methanol. The flavonoid content was calculated and expressed in microgram quercetin per milliliter (*µ*g_quercetin_/mL).

### 2.3. Determination of Scavenging Activities of SCC

The free radical scavenging of SCC at various concentrations (6.25–2,000 *µ*g/mL) was determined by ABTS cation decolorization assay [9] and results were expressed as a percentage of the scavenging. Hydroxyl radical assay according to the Fenton-type reaction was measured. The reaction mixture contained 1 mL of 0.1 mM methyl violet, 0.5 mL of 5 mM FeSO_4_, 0.5 mL of 1% H_2_O_2_, and 2 mL of Tris buffer (pH 4.0). The reaction volume is 10 mL. 0.5 mL of SCC at a different concentration was added. The absorbance of the reaction mixture was measured at 565 nm by a spectrophotometer. The absorbance with SCC was measured as *A*_*s*_, the absorbance without SCC was *A*_0_, and absorbance without FeSO_4_ and H_2_O_2_ was determined as *A*. Scavenging activity of SCC on hydroxyl radical was calculated according to the following formula: [(*A*_*s*_ − *A*_*o*_)/(*A* − *A*_*o*_)] × 100.

The scavenging activity of the SCC on superoxide anion was calculated by comparing Δ*A*_1_/min (without SCC) and Δ*A*_2_/min (with SCC) of the pyrogallol system; 100 *µ*L of 3 mM pyrogallol and 3 mL of Tris buffer (pH 8.2) were mixed with 0.5 mL of SCC at different concentrations and the autooxidation rate of pyrogallol was measured by determining the changes in the absorbance at 325 nm in 4 min by UV-visible spectrophotometer. The absorbance with SCC was *A*_2_ and the absorbance without SCC was determined as *A*_1_ following the formula [(Δ*A*_1_ − Δ*A*_2_)/Δ*A*_1_] × 100 [10]. Ascorbic acid was used as natural antioxidant compound to compare the free radical ABTS^*∙*+^, hydroxyl, and superoxide radical scavenging action with SCC. Nitric oxide scavenging capacity assay was measured at 550 nm using the Griess reagent [11]. Gallic acid was used as a reference of natural antioxidant compound to compare the scavenging action on nitric oxide radical. The scavenging capacities at 50% (SC_50_) of free radical ABTS^o+^, hydroxyl, superoxide, and nitric oxide radicals were calculated.

### 2.4. The Protective Effects of SCC on Acute Gastric Ulcer Induced by Indomethacin* In Vivo*

#### 2.4.1. Animals

The mice were bred at Mahidol University, Bangkok, Thailand. These were procured after obtaining clearance from the respective Animal Committee of the Walailak University, Thailand, and were handled following International Animal Ethics Committee Guidelines. Male mice (25–30 g body weight), five weeks old, were divided into seven groups of six mice each and maintained and kept in controlled environment, at a constant temperature (23 ± 2°C) and humidity (60 ± 10%) and a 12 h light/dark cycle. Mice were acclimatized for one week before any experimental procedures and were allowed standard rat chow and tap water* ad libitum*.

#### 2.4.2. Acute Induction of Indomethacin Gastric Mucosal Lesions and Treatments

According to Santucci et al. [12], mice were conducted with seven independent groups (*n* = 6 per group) and orally treated as follows:

 group 1: untreated control (phosphate buffer solution: PBS),
 group 2: PBS + indomethacin (20 mg/kgBW),
 group 3: SCC (100 mg/kgBW) + indomethacin (20 mg/kgBW),
 group 4: SCC (250 mg/kgBW) + indomethacin (20 mg/kgBW),
 group 5: SCC (500 mg/kgBW) + indomethacin (20 mg/kgBW),
 group 6: omeprazole (100 mg/kgBW) + indomethacin (20 mg/kgBW),
 group 7: vitamin C (100 mg/kgBW) + indomethacin (20 mg/kgBW).

20 mg/kgBW of indomethacin suspended in an aqueous solution of sodium bicarbonate (5%) was administered after one hour of pretreatment of group 2 to group 7. Four hours later, the stomachs of the animals were removed. The ulcerated portions of the stomach were sectioned after fixing in 10% formal saline solution. After 24 h of fixation followed by embedding in a paraffin block, it was cut into sections of 5 *μ*m onto a glass slide; the pathological changes of gastric tissue was evaluated with haematoxylin-eosin staining and examined under a light microscope. The damage scores, inhibition percent, mucin contents, and oxidative and inflammation parameter of all groups were measured.

The gastric ulcer was evaluated by measuring the length of ulcer in mm scale. Ulcer index (UI) was calculated from the total of gastric ulcer area (mm) divided with total mice in each group. Inhibition percent (% inhibition) was calculated following the formula

(1) %Inhibition =UI  of  control  group  −UI  of  treatment  groupUI  of  control  group.  ×100

#### 2.4.3. Determination of Gastric Mucus Content

The mucus bound to the epithelial surface (*μ*g/g tissue) was performed according to the method of Corne et al. [13]. The glandular portion of the stomach was removed and immersed in 0.1% (w/v) alcian blue in sucrose (160 mmol/L) buffered with sodium acetate (50 mmol/L, pH 5.8). The unbound dye was eluted twice with sucrose (250 mmol/L) and the mucus bound dye was extracted with MgCl_2_ (500 mmol/L, pH 6). The solution was then shaken with an equal volume of diethyl ether, centrifuged at 3000 g for 10 min, and the amount of alcian blue in the aqueous phase was measured spectrophotometrically at 580 nm. Pathological study showed higher accurate data more than spectrophotometry.

#### 2.4.4. Determination of Oxidative Damage

(*1) Determination of Lipid Peroxides*. The thiobarbituric acid reaction of Uchiyama and Mihara [14] was adopted for estimation of lipid peroxides level, using malondialdehyde as a standard. Gastric mucosal homogenates 1% (v/v) orthophosphoric acid and 0.6% (w/v) thiobarbituric acid were added; mixtures were then boiled for 45 min at 100°C. After cooling, the colored product was extracted by n-butanol, vortexed, and centrifuged at 3000 g for 15 min. The absorbance of the upper layer was read spectrophotometrically at 535 and 520 nm. The difference in absorbance was calculated as gastric mucosal lipid peroxides level and expressed as nmol/mg tissue malondialdehyde.

(*2) Determination of Oxidized Glutathione (GSSG)*. Oxidized glutathione was determined following Rolland [15]. The gastric tissue was homoginated and diluted to 10 folds; 10 *µ*L of EDTA and 10 *µ*L of NADPH were added and mixed and the baseline level of NADPH absorbance was recorded. For measurement of standard GSSG solution, 1 mL sample was replaced by 1 mL buffer and standard GSSG at volume containing 5 nmol. The oxidized glutathione levels were present as ng/mg protein.

(*3) Determination of Glutathione Peroxidase (GPx).* The reaction mixture consisted of 100 *µ*L of buffer, 20 *µ*L of GSH, 100 *µ*L of GR, 100 *µ*L of NADPH, 10 *µ*L of homoginated gastric tissue, 10 *µ*L of NaN_3_, and 660 *µ*L of distilled water. This mixture was warmed at 37°C for 10 min and then 10 *µ*L of t-butyl hydroperoxide was added. The OD at 340 nm was then followed. A blank value was obtained without any addition of substrates and was subtracted from the assay values. The activity of glutathione peroxidase was calculated from the change in optical density in 1 min and the molar extinction coefficient for NADPH at 340 nm of 6.22 nM/cm. The activity was expressed as U/g protein. Protein (mg/g tissue) was determined by Lowry et al. [16] method.

(*4) Determination of Nitric Oxide Level*. Nitric oxide (nmol/mg tissue) was quantified indirectly as nitrite/nitrate concentration using Griess reaction-dependent method [17]. Gastric mucosal homogenates were deproteinated with absolute ethanol for 48 h at 4°C and then centrifuged at 12000 g for 15 min at 4°C. To an aliquot of the supernatant vanadium trichloride 0.8% (w/v) in 1 M HCl was added for the reduction of nitrate to nitrite, followed by the rapid addition Griess reagent consisting of 0.1% (w/v) N-(1-Naphthyl) ethylenediamine dihydrochloride and 2% (w/v) sulfanilamide in 5% (v/v) HCl and incubated for 30 min at 37°C. Mixtures were cooled and the absorbance at 540 nm was measured.

#### 2.4.5. Determination of Inflammatory Parameter

(*1) Tumor Necrosis Factor-Alpha (TNF-α) Assay.* The plasma samples were collected from the mice. The levels of TNF-*α* in plasma were determined by ELISA assay following the manufacturer's instructions (Millipore, USA). Each sample was added in 96-well plate and incubated for 1 h with TNF-*α* detection antibody solution. Wells were washed 4 times with PBS assay buffer and conjugated with avidin-HRP A solution for 30 min, discarded, and washed to minimize background. 100 *µ*L of substrate solution F was added and incubated for 15 min in the dark to form a complex with a stabilized chromogen. Stop the reaction by adding 100 *µ*L of stop solution to get yellow color. Read absorbance at 450 nm within 30 min and subtract with the absorbance at 570 nm. The TNF-*α* concentration in the sample was calculated from the recombinant mouse TNF-*α* standard curve.

(*2) Western Blot Technique for COX-1, COX-2, and iNOS Expression.* The glandular part of the gastric mucosa, after being washed with PBS containing protease inhibitors, was minced and homogenized in a PBS buffer obtaining protease inhibitor. After centrifugation at 14,000 g for 20 min at 4°C, the supernatant was collected, aliquoted, and kept at −80°C before use in the western blots. The proteins were resolved by SDS-polyacrylamide gel electrophoresis and transferred to a nitrocellulose membrane. The membrane was blocked for 1 h in Tris-buffered saline with Tween 20 (TBST) (20 mM Tris-HCl, pH 7.4, 150 mM NaCl, 0.02% Tween 20) containing 5% bovine serum albumin or skim milk and incubated overnight at 4°C with the appropriate primary antibody. The membrane was washed with TBST twice for 7 min and Tris-buffered saline (TBS). Then membrane was incubated with the appropriate peroxidase conjugated secondary antibody. The bands were detected using an enhanced chemiluminescence detection kit and quantified with respect to the bands of a suitable loading control, using the Kodak Gelquant software [18].

### 2.5. Statistical Analysis

Statistical comparisons were made using one-way ANOVA followed by Newman-Keuls multiple comparisons test. All results are given as mean ± S.E.M. of at least three separate experiments. A value of *P* < 0.05 was considered statistically significant.

## 3. Results

### 3.1. Total Phenolic and Flavonoid Contents of SCC

**Figure 1:**
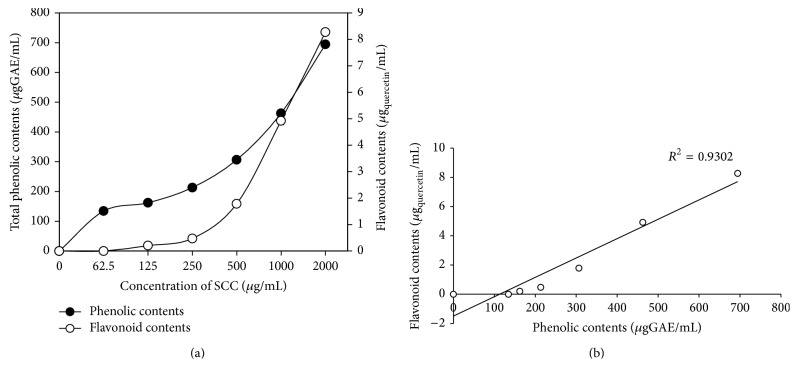
Total phenolic and flavonoid contents of SCC at various concentrations (a) and the correlation of phenolic and flavonoid contents of SCC (b).

Total phenolic content of SCC was found to be increased from 134.44 ± 2.22–694.44 ± 3.64 *µ*gGAE/mL at the concentration of 62.5–2000 *µ*g/mL of SCC, respectively, depending on dose dependent manner. Flavonoid content was calculated from the regression equation of the quercetin calibration curve (*y* = 0.023*X* + 0.118). The total flavonoid content was increased from 0.21 ± 0.04–8.27 ± 0.10 *µ*g_quercetin_/mL at the concentration of 125–2000 *µ*g/mL of SCC, respectively. The result of total phenolic and flavonoid contents are summarized in Figure 1(a) and the correlation between total phenolic and flavonoid contents was almost related and depended on the concentration of SCC at 0.93 of *r* ^2^ value (Figure 1(b)).

### 3.2. Scavenging Activities of SCC

**Figure 2:**
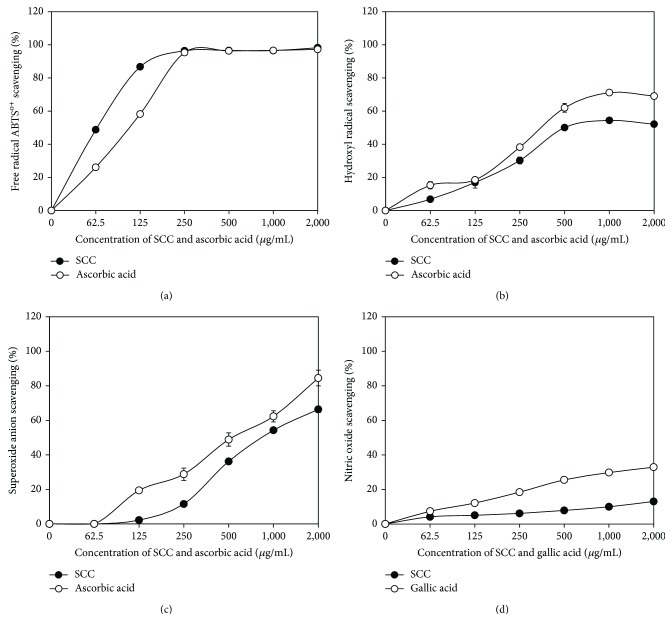
Scavenging activities of SCC, ascorbic acid, and gallic acid on free radical ABTS^∙+^ radical cation (a), hydroxyl radical (b), superoxide radical (c), and nitric oxide radical (d).

Scavenging activities at 50% (SC_50_) values of SCC on free radical ABTS^∙+^, hydroxyl, and superoxide radical were 87.79 ± 0.85, 766.62 ± 28.61, and 1,299 ± 25.13 *µ*g/mL, respectively. This data indicated that increasing of SCC concentration was related to the increased radical scavenging activities, especially for scavenging on ABTS^o+^ radical of SCC was comparable active than ascorbic acid (Figure 2(a)). The scavenging activities of SCC and ascorbic acid at the concentration of 62.5–2,000 *µ*g/mL on hydroxyl radical were increased from 6.81 ± 1.00–52.09 ± 0.75 and 18.29 ± 2.14–69.04 ± 0.97%, respectively (Figure 2(b)) and superoxide radical scavenging activities of SCC and ascorbic acid at the concentration of 125–2,000 *µ*g/mL were concentration dependent increasing from 2.21 ± 0.07–66.36 ± 1.82 and 19.42 ± 1.51–84.50 ± 4.58%, respectively (Figure 2(c)). Nitric oxide scavenging activities of SCC were measured and compared to gallic acid (Figure 2(d)). The scavenging activities of SCC and gallic acid at the concentration of 62.5–2,000 *µ*g/mL on nitric oxide radical were increased from 4.15 ± 0.92–13.05 ± 0.96 and 7.39 ± 0.97–32.89 ± 0.81%, respectively. The SC_50_ of nitric oxide of SCC and gallic acid were not calculated because the scavenge action did not reach 50%; even the concentration of SCC and gallic acid was increased up to 8,000 *µ*g/mL (data not shown).

### 3.3. Protective Effect of SCC on Indomethacin-Induced Gastric Ulceration

#### 3.3.1. Ulcer Index (UI) and Inhibition Percent of SCC Treated Mice

**Figure 3:**
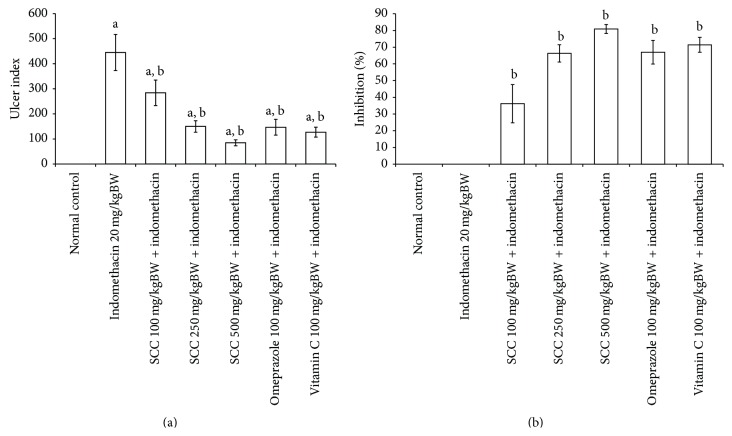
Ulcer index (a) and ulcer inhibition percent (b) of acute study. Data shown as mean ± S.E.M. a: *P* < 0.05 when compared with normal group and b: *P* < 0.05 when compared with indomethacin 20 mg/kgBW group.

The evaluation of gastric ulcer was measured by the length area in mm and calculated into UI values. The UI values of all groups were demonstrated in Figure 3(a). The indomethacin treated group was the highest and showed significant increase in UI values when compared with control (*P* < 0.05) at 445 ± 72.09.

SCC treated group at the concentration of 100–500 mg/kgBW significantly reduced the UI values when compared with indomethacin treated group and UI values of SCC at 100–500 mg/kgBW were 284.09 ± 50.82, 150 ± 22.82, and 85 ± 11.90, respectively. The SCC at 500 mg/kgBW was more potent than other compounds; it has shown about 80% decrease in UI when compared to indomethacin treated group.

Omeprazole and vitamin C at 100 mg/kgBW significantly decreased the UI values when compared with indomethacin treated group and had shown the UI values at 146.88 ± 31.49 and 127.27 ± 19.70, respectively.

**Figure 4:**
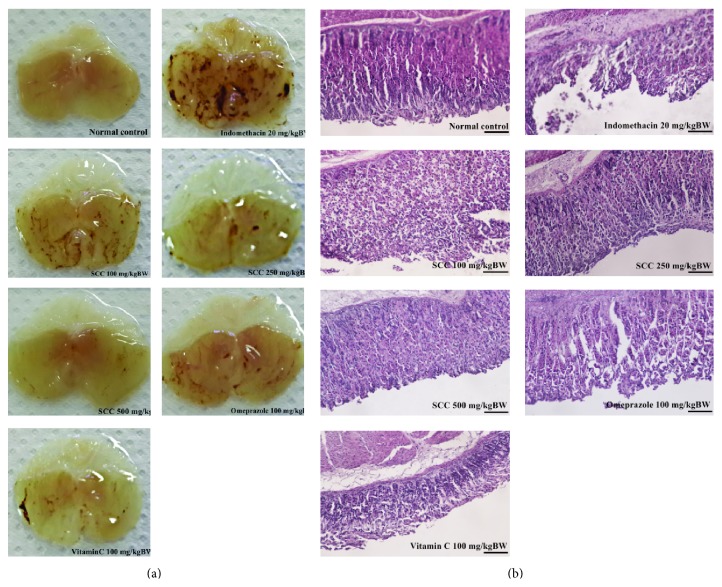
Photograph (a) and histology (b) of protective effect of SCC on gastric tissue after being induced with 20 mg/kgBW of indomethacin. Dark spot represented the ulcer area in gastric tissue and black bars represent size of tissue at 10 *µ*m.

UI values were calculated into inhibition percent by being compared with indomethacin treated group. SCC treated group at the concentration of 100–500 mg/kgBW inhibited the gastric ulcer at 36.16 ± 11.42, 66.29 ± 5.13, and 80.89 ± 2.67%, respectively (Figure 3(b)). Omeprazole and vitamin C at 100 mg/kgBW inhibited the ulceration by induction of the inhibition percent to 66.99 ± 7.08 and 71.39 ± 4.45%, respectively. The SCC, omeprazole, and vitamin C treated groups significantly increased inhibition percent when being compared with indomethacin treated group (*P* < 0.05). SCC at 500 mg/kgBW was the highest effective compound to protect the ulceration induced by 20 mg/kgBW indomethacin and significantly reduced the ulcer better than omeprazole and vitamin C treated group. The physiology of gastric tissue of each group was demonstrated in Figure 4(a). The dark areas in the photograph represented the area of ulceration. The indomethacin treated group had shown the severe ulcer area with many dark spot areas. However, when mice were treated with SCC, omeprazole, and vitamin C, the areas of ulcer were reduced in all groups. Specifically, when treated with SCC 500 mg/kgBW, the physiology of gastric tissue is not different from normal mice.

#### 3.3.2. Pathology of Gastric Tissue of SCC Prevents Gastric Ulceration Induced by Indomethacin

Figure 4(b) demonstrated the pathology of gastric tissue after being treated with indomethacin, SCC 100–500 mg/kgBW, omeprazole, and vitamin C. The normal control group showed intact mucosal epithelium, submucosa, and muscularis mucosa. Indomethacin treated group had shown large erosion area in the mucosal epithelium with presence of crypts; ulcer pits and damage muscularis mucosal layer were observed. Treatment with SCC can protected the gastric tissue from the action of indomethacin damage cell. At the concentration of 100 and 250 mg/kgBW of SCC treated group the rupture at various portions of the mucosal epithelial layer was presented and the crypts in the mucosal glandular layer and slightly disrupted muscle layer similar results to omeprazole and vitamin C treatment group were showed. SCC at 500 mg/kgBW treated groups significantly reduced the damage of gastric cell and protected gastric cell as well as the normal control group.

#### 3.3.3. Determination of Gastric Wall Mucus in Indomethacin-Induced Acute Gastric Ulceration

The alcian blue-binding capacity, which indicates the acidic mucopolysaccharide content of gastric mucin of normal control, indomethacin, SCC 100–500 mg/kgBW, omeprazole, and vitamin C 100 mg/kgBW treated groups were 19.35 ± 0.94, 15.78 ± 0.33, 20.74 ± 0.75, 21.13 ± 0.98, 23.59 ± 0.55, 21.31 ± 0.75, and 16.29 ± 0.64 *µ*g alcian blue/g wet stomach, respectively. After mice were treated with PBS 1 h before giving indomethacin at 20 mg/kgBW mucus content was significantly decreased when compared to normal mice. In contrast, SCC 100–500 and omeprazole 100 mg/kgBW significantly increased mucus levels compared to indomethacin treated group which indicated the defensive system of SCC extract and omeprazole against indomethacin induced gastric ulcer. However, we found that vitamin C treated group showed no difference of mucus content compared to indomethacin treated alone group (Table 1).

#### 3.3.4. Determination of SCC Inhibited Oxidative Damage on Indomethacin-Induced Acute Gastric Ulceration

The lipid peroxidation, GSSG, GPx, and NO were demonstrated in Table 1.

(*1) Effect on Lipid Peroxidation.* The MDA levels were increased markedly after being treated with 20 mg/kgBW of indomethacin in either plasma or gastric tissue when compared to normal mice (*P* < 0.05). In plasma treatment with SCC 100–500 mg/kgBW reduced the MDA levels compared with indomethacin treated group. The concentration of SCC at 250 and 500 mg/kgBW were significantly improved MDA levels better than omeprazole treated group (*P* < 0.05) and were not different from the MDA levels of untreated control mice.

MDA levels in gastric tissue were similar to plasma MDA levels in which pretreatment of PBS before 20 mg/kgBW of indomethacin significantly increased the MDA level (*P* < 0.05) when compared to normal mice while SCC treated groups decreased the levels of MDA in a dose dependent manner. SCC at 100–500 mg/kgBW significantly decreased MDA when compared with indomethacin treated group (*P* < 0.05) and maintained the level as well as normal mice. Omeprazole and vitamin C at 100 mg/kgBW also significantly reduced MDA levels compared to indomethacin treated group in gastric tissue.

(*2) Effect on Oxidized Glutathione.* Mice in indomethacin treated group showed a higher level of GSSG compared to normal mice. In contrast, the levels of GSSG were decreased in the SCC, omeprazole, and vitamin C compared to indomethacin treated group. The levels of GSSG in SCC at 500 mg/kgBW were reduced nearly to the GSSG level of normal mice.

(*3) Effect on Glutathione Peroxidase (GPx).* The GPx activity in gastric tissue was based on measurement of the oxidation of NADPH. The activities of the antioxidant enzymes GPx were significantly decreased in indomethacin treated mice compared to normal mice group. Treatment of SCC, omeprazole, and vitamin C before treatment with 20 mg/kgBW indomethacin increased the activities of GPx compared to indomethacin treated group. Treatment of SCC at 500 mg/kgBW protected the gastric mucosal against the loss of antioxidant enzyme activity, resulting in a significant increase in enzymatic GPx activities approximate to the normal control levels.

(*4) Effect on Nitrite Contents.* The nitrite content of indomethacin treated group was significantly increased compared to the normal control group (*P* < 0.05), while the nitrite content of the SCC, omeprazole, and vitamin C was significantly lower than that in the indomethacin treated group.

#### 3.3.5. The Effect of SCC on Inflammation Parameter

**Figure 5:**
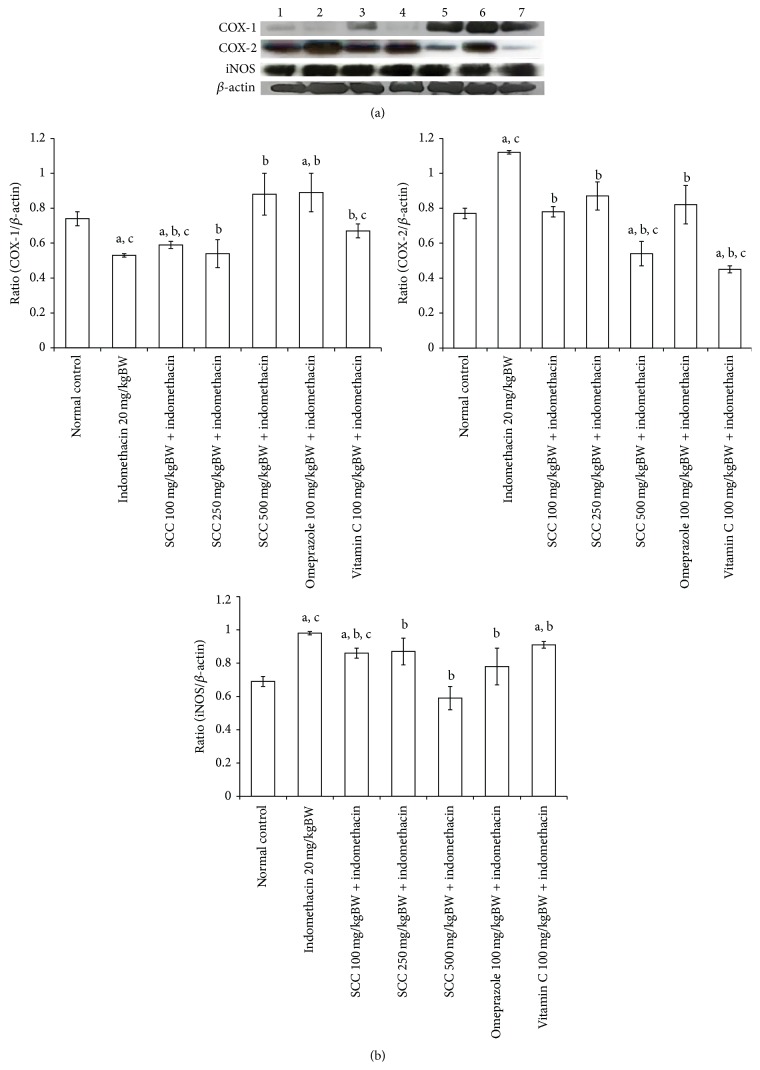
COX-1, COX-2, and iNOS expression in gastric tissue of acute study. Western blot was represented, showing the ratio of COX-1, COX-2, and iNOS compared to *β*-actin band ((a) and (b)). Analysis represents mean ± S.E.M. a: *P* < 0.05 compared with normal mice, b: *P* < 0.05 compared with indomethacin treated group, and c: *P* < 0.05 compared with omeprazole treated group.

(*1) Plasma TNF-α.* Plasma TNF-*α* level of indomethacin treated group was at 2.09 ± 0.13 pg/mL and was significantly increased when compared to normal mice (*P* < 0.05). Pretreatment of SCC at concentration of 500 mg/kgBW or vitamin C showed significantly reduced TNF-*α* levels compared to indomethacin treated group (*P* < 0.05), while omeprazole treated group was not significantly different compared to indomethacin treated group (Table 1).

(*2) The Expression of COX-1, COX-2, and iNOS.* Figure 5 showed the expression of COX-1, COX-2, and iNOS on SCC protective effect on indomethacin-induced gastric ulcer. The expressing of COX-1 was reduced after 20 mg/kgBW indomethacin administration and COX-2 was increased in the same group. The iNOS protein levels in indomethacin treated mice indicated upregulated iNOS gene expression. However, treatment with SCC significantly reduced the expression of this enzyme (*P* < 0.05). Thus, SCC, omeprazole, and vitamin C which reduced the expression of iNOS enzyme level led to the decrease of the NO level which further prevented the acute phase inflammation and regulation of the severity of gastric ulcer.

## 4. Discussion

*Syzygium cumini* (L.) Skeels leaves contain various medicinal compounds and were used for treatment in various pharmaceutical preparations. It is well-known that natural phenolic compounds contribute to quality and nutritional value in terms of modifying color, taste, aroma, and flavor and also in providing health benefit effects. The natural phenolic compounds also serve in plant as defense mechanisms to counteract ROS in order to survive and prevent molecular damage and damage by microorganisms, insect, and herbivores [19]. Total phenolic compounds, including tannins and flavonoids, have been reported to have multiple biological properties to possess general antimicrobial and antioxidant activity [20, 21]. The strong antioxidant activity of the SCC may be attributed to the phenolic compound and flavonoid content which were correlated with *r* ^2^ value at 0.93. In this study, SCC scavenge free radical ABTS^∙+^, hydroxyl, superoxide anion, and nitric oxide radical* in vitro* indicated that the SCC may help to arrest the chain of reactions initiated by excess generation of OH^∙^, O_2_^∙^, and NO^∙^ that similarly reported the effectiveness in human health [21]. SCC also showed no pathological side effect on tissues damage in both liver and kidney tissue.

Indomethacin possesses the highest ulcerogenic potential to humans among the commonly used NSAIDs. The gastric damage induced by indomethacin in mice can be attributed to their ability to induce the reactive oxygen metabolites. The notion that SCC showed powerful* in vitro* antioxidant property encouraged us to investigate their possible protective effect against indomethacin NSAIDs induced gastric ulcer. The induction of gastric ulceration by indomethacin in acute model was associated with gastric lipid peroxidation and inflammatory reaction to produced ROS which leading to severe ulceration on gastric tissue and were demonstrated on UI values, photograph, and pathology of gastric mucosa of this work. The reduction of gastric ulcer area found in SCC treated group according to their ROS scavenge resulted in increasing inhibitory percent.

The gastric mucosal barrier is a complex system made up of submucosal epithelial and mucus elements [22]. The mucus gel layer is a thick organized layer adherent to the epithelium and plays an important role in protection of the epithelium against acid, pepsin, and mechanical damage [23, 24]. The stimulation of production of gastric mucin, PGE_2_, and bicarbonate and the decrease of acid output help to maintain mucosal integrity. An increase in mucus production usually assists the healing process by protecting the ulcer crater against the endogenous aggressors like stomach secretions and oxidants as well as against exogenous damaging agents such as NSAIDs. These results revealed that stomach ulceration in indomethacin treated group resulted in decreased mucin secretion. This might reduce the ability of the mucosal membrane to protect the mucosa from physical damage and back diffusion of hydrogen ions. Apparently, the antioxidative property of the SCC might be contributing in protecting the oxidative damage to gastric mucosa.

Tissue damage is always associated with excess generation of ROS leading to oxidative stress. ROS mediated lipid peroxidation is one of the principle causes of gastrointestinal lesion induced by indomethacin [25]. The free radicals, in particular hydroxyl radical, begin the peroxidation of cell membrane, causing the liberation of arachidonic acid and peroxyl lipid free radical [26]. The peroxyl radical promotes additional lipid peroxidation, removing hydrogen from fatty acid and originating a chain reaction in which the final product is the MDA [27].

SCC was corroborated by preventing the indomethacin-induced lipid peroxidation. The ROS-mediated degradation of cell membrane results in the formation of lipid peroxides and initiates a variety of deleterious sequence, including mucosal lesions and depletion of mucus layer, alterations that were confirmed in this work in indomethacin treated group. The gastroprotection of SCC depends also on replenishing the increased GSSG. The endogenous defense molecule maintains the normal redox potential and counteracts free radical-or-toxic induced damage including superoxide dismutase (SOD) and glutathione peroxidase (GPx) [28, 29]. Indeed, our work showed that SCC leveled up GPx activity above the normal level and hence improves the endogenous defense system, which was demolished by the indomethacin direct inhibitory effect on peroxidases [30] and hydrogen peroxide overproduction by the gastric mucosa [31]. The high activity may explain the prevention of lipid peroxidation by SCC, especially in the presence of normal level of its product, GSSG.

Nitric oxide (NO) is a crucial mediator of gastrointestinal mucosal defense but it also contributes to mucosal damage [32]. This can be illustrated by the ability of different nitric oxide concentration to produce completely opposite effects in the same tissue [33]. There is evidence that low doses of NO releasing substances protect against NSAID induced gastropathy and increase the healing rate of gastric ulcers. However, high doses of these substances could induce extensive hemorrhagic mucosal damage [34]. NO is produced by NOsynthase (NOS) in various types of cells, including endothelial cells, neurons, neutrophils, macrophages, and brush cells of the gastric surface epithelium [35]. There is growing evidence that the major source of ROS is from the activated neutrophils [36] and neutrophils play a vital role in the development of gastric damage by their aggregation and release of tissue disturbing substance such as oxygen free radicals and proteases [37]. Therefore, the NO levels of indomethacin treated group in acute study were increased due to the directly stimulated NO from neutrophils and macrophages. Thus, SCC reduced the nitrite concentration compared to indomethacin treated group by their scavenger property.

TNF-*α* seems to be one of the key factors for several forms of mucosal gastric lesions including NSAIDs. The plasma levels of TNF-*α* have been observed to increase markedly after administration of NSAIDs. Also, TNF-*α* plays a critical role in NSAIDs induced gastric injury by modulating neutrophil infiltration [38] and induces leucocytes adherence after indomethacin administration [18]. Thus, SCC inhibited the increase production of proinflammatory cytokines, TNF-*α*, through its antioxidant effects.

NSAIDs inhibit prostaglandin (PG) production by inhibiting COX. Two isoforms of COX enzyme are known to be involved in PG synthesis. COX-1 is constitutively expressed and generates PG involved in gastrointestinal protection and platelet function, while at site of inflammation, COX-2 is induced to generate PG which mediates inflammation and pain [39]. The NSAIDs like indomethacin exert both their therapeutic and toxic effects mainly through inhibiting cyclooxygenases and decreasing the levels of circulating PGE_2_ at gastric mucosa causing gastric ulcerations and also exacerbating preexisting gastric ulcers in rodent and humans [40]. Indomethacin has a weak selectivity for COX-1 and we can argue that the inhibition of COX-2 by intravenously administered indomethacin was not enough, thereby resulting in the development of gastric lesions. The present report shows a reduction of COX-1 was expressed after 20 mg/kgBW indomethacin administration and COX-2 was increased in the same group. This finding can explain that the high doses of indomethacin was induced gastric lesions than to inhibited COX-2 expression on the gastric mucosa, because higher concentrations were required for inducing the direct cytotoxic effect of NSAIDs than were required for inhibiting PG synthesis [41]. Therefore, the compound which is selective for COX-2 should theoretically provide efficacy comparable to traditional nonselective NSAIDs without inducing the gastrointestinal injury or platelet dysfunction as in our studies; SCC stimulated the expression of COX-1 and inhibited the expression of COX-2 by dose dependent manner.

Omeprazole is one of drugs used for the prevention of gastrointestinal side effects of NSAIDs. In previous study, omeprazole provided more effective protection than placebo in the healing of both gastric and duodenal mucosa damage [42, 43]. The protective effects of pretreatment with omeprazole are compared to SCC in indomethacin treated mice. Omeprazole had significant protection effects compared to indomethacin treated group but the prevention effect of omeprazole was less than SCC, may be due to the ROS scavenging action of natural compounds. Similar to our study, it has been reported that omeprazole decreased the production of gastric ulcers by more than 80% [44]. However, in terms of drug usage, combination of omeprazole and SCC could be rise up the prevention processes through breaking ROS and proton pump action in gastric ulcer and reduced the cost on treatment processes. Vitamin C was used as a reference antioxidant compound to support the antioxidant action of SCC on indomethacin-induced gastric ulcer. However, the high dose of vitamin C (100 mg/kgBW) in our study did not show the good protection in indomethacin-induced acute ulceration when compared to SCC and omeprazole treated groups.

## 5. Conclusions

SCC has shown an excellent in* in vitro* and* in vivo* antioxidant and ROS scavenging activity and has been established as a natural antioxidant. SCC has shown the strong action in antioxidant and anti-inflammatory activities. Furthermore, SCC is action as anti-inflammatory compounds by breaking of NO, TNF-*α* in inflammatory process, reduced the inhibition of COX-1 and drop downed the expression of COX-2, resulting in the reduction of severe damage of gastric cells from indomethacin action. From this powerful action of SCC, we can summarize that, SCC can be used as anti-inflammation and antigastric ulcer compound without toxicity.

## Figures and Tables

**Table 1:** Malondialdehyde (MDA), oxidized glutathione (GSSG), glutathione peroxidase (GPx), nitrite contents, gastric wall mucus, and plasma TNF-*α* in gastric tissue of SCC acute test group.

Parameter	Group 1	Group 2	Group 3	Group 4	Group 5	Group 6	Group 7
*Mucus* (*µ*g Alcian blue/g wet stomach)	19.35 ± 0.94	15.78 ± 0.33^a,c^	20.74 ± 0.75^b^	21.13 ± 0.98^b^	23.59 ± 0.55^a,b,c^	21.31 ± 0.75^a,b^	16.28 ± 0.64^a,c^

*MDA *							
Plasma (nM/mL)	17.97 ± 2.69	23.99 ± 2.49^a,c^	16.54 ± 5.53^b^	16.45 ± 4.04^b,c^	16.01 ± 3.19^b,c^	19.06 ± 2.35^a,b^	19.17 ± 2.55^a,b^
Tissue (nM/mg tissue)	1.18 ± 0.11	1.55 ± 0.03^a,c^	1.07 ± 0.29^b^	1.03 ± 0.12^b^	0.92 ± 0.04^a,b^	1.04 ± 0.14^b^	1.29 ± 0.06^a,b,c^

*GSSG* (ng/mg Protein)	0.75 ± 0.01	1.26 ± 0.04^a,c^	0.99 ± 0.04^a,b^	0.87 ± 0.05^a,b,c^	0.81 ± 0.01^a,b,c^	0.98 ± 0.04^a,b^	0.87 ± 0.01^a,b,c^

*GPx* (U/g Protein)	2.41 ± 0.37	0.75 ± 0.19^a,c^	1.17 ± 0.13^a,b,c^	1.19 ± 0.16^a,b,c^	2.59 ± 0.13^b,c^	0.99 ± 0.05^a,b^	0.89 ± 0.04^a,b,c^

*Nitric oxide* (nM/mg Protein)	1.02 ± 0.31	2.39 ± 0.49^a,c^	1.27 ± 0.311^b,c^	1.07 ± 0.37^b,c^	0.68 ± 0.23^a,b,c^	1.54 ± 0.18^a,b^	0.78 ± 0.11^b,c^

*Plasma TNF*-*α* (pg/mL)	1.71 ± 0.04	2.09 ± 0.13^a^	2.07 ± 0.09^a,c^	1.92 ± 0.05^a^	1.83 ± 0.03^a,b^	1.89 ± 0.05^a^	1.80 ± 0.06^a,b^

^a^ *P* < 0.05 compared with normal mice group, ^b^ *P* < 0.05 when compared with indomethacin treated group, and ^c^ *P* < 0.05 when compared with omeprazole treated group.

## References

[1] Braga F. G., Bouzada M. L. M., Fabri R. L. (2007). Antileishmanial and antifungal activity of plants used in traditional medicine in Brazil. *Journal of Ethnopharmacology*.

[2] Pepato M. T., Mori D. M., Baviera J. B., Harami R. C., Vendramini R., Brunetti I. L. (2004). Fruit of the jambolan tree (*Eugenia jambolana* Lam.) and experimental diabetes. *Journal of Ethnopharmacology*.

[3] Ayyanar M., Subash-Babu P. (2012). *Syzygium cumini* (L.) skeels: a review of its phytochemical constituents and traditional uses. *Asian Pacific Journal of Tropical Biomedicine*.

[4] Polat B., Suleyman H., Alp H. H. (2010). Adaptation of rat gastric tissue against indomethacin toxicity. *Chemico-Biological Interactions*.

[5] Osborn L. (1990). Leukocyte adhesion to endothelium in inflammation. *Cell*.

[6] Biswas K., Bandyopadhyay U., Chattopadhyay I., Varadaraj A., Ali E., Banerjee R. K. (2003). A novel antioxidant and antiapoptotic role of omeprazole to block gastric ulcer through scavenging of hydroxyl radical. *Journal of Biological Chemistry*.

[7] Lister E., Wilson P. (2001). *Measurement of Total Phenolics and ABTS Assay for Antioxidant Activity (Personal Communication)*.

[8] Chang C.-C., Yang M.-H., Wen H.-M., Chern J.-C. (2002). Estimation of total flavonoid content in propolis by two complementary colometric methods. *Journal of Food and Drug Analysis*.

[9] Re R., Pellegrini N., Proteggente A., Pannala A., Yang M., Rice-Evans C. (1999). Antioxidant activity applying an improved ABTS radical cation decolorization assay. *Free Radical Biology and Medicine*.

[10] Sun D., Zhang S., Wei Y., Yin L. (2009). Antioxidant activity of mangostin in cell-free system and its effect on K562 leukemia cell line in photodynamic therapy. *Acta Biochimica et Biophysica Sinica*.

[11] Hoque N., Imam M. Z., Akter S. (2011). Antioxidant and antihyperglycemic activities of methanolic extract of *Glinus oppositifolius* leaves. *Journal of Applied Pharmaceutical Science*.

[12] Santucci L., Fiorucci S., Giansanti M., Brunori P. M., Di Matteo F. M., Morelli A. (1994). Pentoxifylline prevents indomethacin induced acute gastric mucosal damage in rats: role of tumour necrosis factor alpha. *Gut*.

[13] Corne S. J., Morrissey S. M., Woods R. J. (1974). A method for the quantitative estimation of gastric barrier mucus. *The Journal of Physiology*.

[14] Uchiyama M., Mihara M. (1978). Determination of malonaldehyde precursor in tissues by thiobarbituric acid test. *Analytical Biochemistry*.

[15] Rolland R. M. (2000). A review of chemically-induced alterations in thyroid and vitamin A status from field studies of wildlife and fish. *Journal of Wildlife Diseases*.

[16] Lowry O. H., Nira J., Rosebrough A., Lewis F., Rose J. R. (1951). Folin phenol reagent protein measurement. *The Journal of Biological Chemistry*.

[17] Miranda K. M., Espey M. G., Wink D. A. (2001). A rapid, simple spectrophotometric method for simultaneous detection of nitrate and nitrite. *Nitric Oxide: Biology and Chemistry*.

[18] Yadav S. K., Adhikary B., Chand S., Maity B., Bandyopadhyay S. K., Chattopadhyay S. (2012). Molecular mechanism of indomethacin-induced gastropathy. *Free Radical Biology and Medicine*.

[19] Vaya J., Belinky P. A., Aviram M. (1977). Antioxidant constituents from licorice roots: isolation, structure elucidation and antioxidative capacity toward LDL oxidation. *Free Radical Biology & Medicine*.

[20] Rivière C., Hong V. N. T., Pieters L. (2009). Polyphenols isolated from antiradical extracts of *Mallotus metcalfianus*. *Phytochemistry*.

[21] Kumar V., Singh P., Chander R. (2009). Hypolipidemic activity of *Hibiscus rosa sinensis* root in rats. *Indian Journal of Biochemistry & Biophysics*.

[22] Allen A., Flemstrom G., Garner A., Kivilaakso E. (1993). Gastroduodenal mucosal protection. *Physiological Reviews*.

[23] Seno K., Joh T., Yokoyama Y., Itoh M. (1995). Role of mucus in gastric mucosal injury induced by local ischemia/reperfusion. *Journal of Laboratory and Clinical Medicine*.

[24] Kawai T., Joh T., Iwata F., Itoh M. (1994). Gastric epithelial damage induced by local ischemia-reperfusion with or without exogenous acid. *The American Journal of Physiology*.

[25] Naito Y., Yoshikawa T., Yoshida N., Kondo M. (1998). Role of oxygen radical and lipid peroxidation in indomethacin-induced gastric mucosal injury. *Digestive Diseases and Sciences*.

[26] Yoshikawa T., Ueda S., Naito Y. (1989). Role of oxygen-derived free radicals in gastric mucosal injury induced by ischemia or ischemia-reperfusion in rats. *Free Radical Research Communications*.

[27] Ribeiro M. E., Yoshida W. B. (2005). Lesoes intestinais decorrentes de isquemiareperfusao: fisiopatologia e modelos experimentais. *Jornal Vascular Brasileiro*.

[28] Loguercio C., Di Pierro M. (1999). The role of glutathione in the gastrointestinal tract: a review. *Italian Journal of Gastroenterology and Hepatology*.

[29] Pastoris O., Verri M., Boschi F. (2008). Effects of esomeprazole on glutathione levels and mitochondrial oxidative phosphorylation in the gastric mucosa of rats treated with indomethacin. *Naunyn-Schmiedeberg's Archives of Pharmacology*.

[30] Banerjee R. K. (1990). Nonsteroidal anti-inflammatory drugs inhibit gastric peroxidase activity. *Biochimica et Biophysica Acta*.

[31] Hassan A., Martin E., Puig-Parellada P. (1998). Role of antioxidants in gastric mucosal damage induced by indomethacin in rats. *Methods and Findings in Experimental and Clinical Pharmacology*.

[32] Muscará M. N., Wallace J. L. (1999). Nitric oxide v. therapeutic potential of nitric oxide donors and inhibitors. *The American Journal of Physiology—Gastrointestinal and Liver Physiology*.

[33] Wallace J. L., Miller M. J. S. (2000). Nitric oxide in mucosal defense: a little goes a long way. *Gastroenterology*.

[34] Souza M. H. L. P., Paula Lemos H., Oliveira R. B., Cunha F. Q. (2004). Gastric damage and granulocyte infiltration induced by indomethacin in tumour necrosis factor receptor 1 (TNF-R1) or inducible nitric oxide synthase (iNOS) deficient mice. *Gut*.

[35] Noda A., Nakata S., Koike Y. (2007). Continuous positive airway pressure improves daytime baroreflex sensitivity and nitric oxide production in patients with moderate to severe obstructive sleep apnea syndrome. *Hypertension Research*.

[36] Pan Q., Shai O., Leo J. L., Brendan J. F., Benjamin J. B. (2008). Deep surveying of alternative splicing complexity in the human transcriptome by high-throughput sequencing. *Nature Genetics*.

[37] Kobayashi T., Ohta Y., Yoshino J., Nakazawa S. (2001). Teprenone promotes the healing of acetic acid-induced chronic gastric ulcers in rats by inhibiting neutrophil infiltration and lipid peroxidation in ulcerated gastric tissues. *Pharmacological Research*.

[38] Appleyard C. B., McCafferty D.-M., Tigley A. W., Swain M. G., Wallace J. L. (1996). Tumor necrosis factor mediation of NSAID-induced gastric damage: role of leukocyte adherence. *The American Journal of Physiology—Gastrointestinal and Liver Physiology*.

[39] Laine L., Bombardier C., Hawkey C. J. (2002). Stratifying the risk of NSAID-related upper gastrointestinal clinical events: results of a double-blind outcomes study in patients with rheumatoid arthritis. *Gastroenterology*.

[40] Wallace J. L. (1997). Nonsteroidal anti-inflammatory drugs and gastroenteropathy: the second hundred years. *Gastroenterology*.

[41] Tomisato W., Tsutsumi S., Hoshino T. (2004). Role of direct cytotoxic effects of NSAIDs in the induction of gastric lesions. *Biochemical Pharmacology*.

[42] Cooke C. E. (1996). Disease management: prevention of NSAID-induced gastropathy. *Drug Benefit Trends*.

[43] Izzettin F. V., Sancar M., Okuyan B., Apikoglu-Rabus S., Cevikbas U. (2012). Comparison of the protective effects of various antiulcer agents alone or in combination on indomethacin-induced gastric ulcers in rats. *Experimental and Toxicologic Pathology*.

[44] Lee M., Kallal S. M., Feldman M. (1996). Omeprazole prevents indomethacin-induced gastric ulcers in rabbits. *Alimentary Pharmacology and Therapeutics*.

